# Altered brain network functional connectivity patterns in patients with vestibular migraine diagnosed according to the diagnostic criteria of the Bárány Society and the International Headache Society

**DOI:** 10.1007/s00415-021-10868-0

**Published:** 2021-11-18

**Authors:** Zhe-Yuan Li, Li-Hong Si, Bo Shen, Xu Yang

**Affiliations:** Department of Neurology, Aerospace Center Hospital, Peking University Aerospace School of Clinical Medicine, Beijing, 100049 People’s Republic of China

**Keywords:** Vestibular migraine, Resting-state functional magnetic resonance imaging, Independent component analysis, Auditory network, Functional connectivity

## Abstract

**Background:**

Vestibular migraine (VM) is considered one of the most common causes of episodic central vestibular disorders, the mechanism of VM is currently still unclear. The development of functional nuclear magnetic resonance (fMRI) in recent years offers the possibility to explore the altered functional connectivity patterns in patients with VM in depth. The study aimed to investigate altered patterns of brain network functional connectivity in patients with VM diagnosed based on the diagnostic criteria of the Bárány Society and the International Headache Society, and hope to provide a scientific theoretical basis for understanding whether VM is a no-structural central vestibular disease, i.e., functional central vestibular disease with altered brain function.

**Methods:**

Seventeen patients with VM who received treatment in our hospital from December 2018 to December 2020 were enrolled. Eight patients with migraine and 17 health controls (HCs) were also included. Clinical data of all patients were collected. Blood pressure, blood routine tests and electrocardiography were conducted to exclude other diseases associated with chronic dizziness. Videonystagmography, the vestibular caloric test, the video head impulse test and vestibular-evoked myogenic potentials were measured to exclude peripheral vestibular lesions. MRI was utilized to exclude focal lesions and other neurological diseases. All subjects underwent fMRI. The independent component analysis was performed to explore changes in intra- and inter-network functional connectivity in patients with VM.

**Results:**

Among 17 patients with VM, there were 7 males and 10 females with an average age of 39.47 ± 9.78 years old. All patients had a history of migraine. Twelve (70.6%) patients had recurrent spontaneous vertigo, 2 (11.7%) patients had visually induced vertigo, and 3 (17.6%) patients had head motion-induced vertigo. All 17 patients with VM reported worsening of dizziness vertigo during visual stimulation. The migraine-like symptoms were photophobia or phonophobia (*n* = 15, 88.2%), migraine-like headache (*n* = 8, 47.1%), visual aura during VM onset (*n* = 7, 41.2%). 5 (29.4%) patients with VM had hyperactive response during the caloric test, and 12 (70.6%) patients had caloric test intolerance. Eleven (64.7%) patients had a history of motion sickness. Totally 13 independent components were identified. Patients with VM showed decreased functional connectivity in the bilateral medial cingulate gyrus and paracingulate gyrus within sensorimotor network (SMN) compared with HCs. They also showed weakened functional connectivity between auditory network (AN) and anterior default mode network (aDMN) compared with HCs, and enhanced functional connectivity between AN and the salience network (SN) compared with patients with migraine.

**Conclusion:**

Patients with vestibular migraine showed obvious altered functional connectivity in the bilateral medial cingulate gyrus and paracingulate gyrus within the SMN. The median cingulate and paracingulate gyri may be impaired, the disinhibition of sensorimotor network and vestibular cortical network may result in a hypersensitivity state (photophobia/phonophobia). Altered functional connectivity between AN and DMN, SN may lead to increased sensitivity to vestibular sensory processing.

## Introduction

Vestibular migraine (VM) is considered the most common cause of central episodic vertigo, affects more than 1% of the general population [1], 7% of patients seen in neuro-otological clinics and 9% of patients seen in headache clinics [2]. At present, the diagnosis of VM is only based on the history of vertigo and migraine, and there is still a lack of more accurate laboratory tests and objective imaging evidence that supports the diagnosis. The underlying mechanisms of VM are still unclear. Many theories have been proposed to explain the pathogenesis of VM, including cortical spreading depression, transmitter/vascular/inflammatory mechanism, genetic predisposition, and functional brain changes [3]. The application of functional imaging techniques, such as positron emission tomography (PET) and functional nuclear magnetic resonance (fMRI), provides a possibility to explore the involvement of functional brain changes in the pathogenesis of VM.

In recent years, the mechanisms underlying the abnormal integration of the central vestibular network in patients with VM have received more attention from researchers. Shin et al. [4] performed 8F-deoxyglucose PET study in two patients with VM during the inter-ictal and ictal period, and found that compared with patients with VM during inter-ictal period, patients with VM during the ictal period showed increased metabolism in the bilateral cerebellum, frontal cortices, temporal cortex, posterior insula, and thalami, and decreased metabolism in the occipital cortex; increased metabolism in the temporo-parieto-insular areas and bilateral thalami indicates the activation of the vestibulo-thalamo-cortical pathway, decreased metabolism in the occipital cortex suggests reciprocal inhibition between the visual and vestibular systems. These findings revealed that metabolic activities in the visual and vestibular cortical areas were altered during acute VM attack. In previous studies, scholars have used task fMRI data from different tasks to investigate VM. Russo et al. [5] performed fMRI study in 12 patients with VM during ear irrigation with cold water and found that patients with VM showed significantly increased activation in the left dorsal thalamus during left-sided vestibular stimulation, and the authors speculated that the pain and vestibular senses in patients with VM may be related to the thalamus. Although these findings may suggest the involvement of supra-tentorial vestibular structures in the occurrence and development of VM, the origin of vestibular dysfunction remains to be elucidated. Structural-based MRI studies are quite heterogeneous. MRI study using voxel-based morphometry (VBM) conducted by Obermann et al. [6] revealed decreased gray matter (GM) volume in the bilateral inferior temporal gyri, cingulate gyrus and posterior insula, as well as in the superior and middle temporal gyri, the superior and inferior occipital gyri, superior parietal lobule and the dorsolateral prefrontal cortex. These areas are associated with higher-level multisensory integration of vestibular information. Messina et al. [7] documented increased GM volume in the left superior occipital gyrus, thalamus, middle temporal gyrus, and right subgenual cingulate gyrus in patients with VM. In fact, there is heterogeneity among the above-mentioned two studies, the reasons may be that: (1) these two studies included different subjects, VM patients with VM who had peripheral vestibular disorder were excluded from the study of Obermann et al. [8], but were included in the study of Messina et al. [7]; (2) although the mean disease duration in patients with VM included in these two studies was approximately 6 years, patients with VM in the study of Messina et al. [7] had a much wider range of disease duration than in the study of Obermann et al. [6] (0.1–23 years vs 1–15 years); (3) patients in these two studies were treated with different types of drugs. Most patients with VM in the study of Obermann et al. [6], were treated with non-steroidal analgesics, and in the study of Roberta Messina et al. [7], patients were treated with Ginkgolide B. Ginkgolide B is a platelet-activating factor antagonist that can increase cerebral cortex blood oxygen supply; this is possibly associated with increased GM volume in cortical lesion areas. The findings of these two studies both suggest that an abnormal brain sensitization might lead to a dis-modulation of multimodal sensory integration and processing cortical areas in patients with VM.

At present, fMRI studies on patients with VM are mostly task- and structural- based, and most studies showed that activation of multi-level vestibular integrating centers and cortical areas related to higher-level vestibular information processing is present in patients with VM. However, task-related brain regions are sometimes not associated with brain functional changes related to VM itself, which may be associated with brain functional changes related to task stimulation [8]. The human brain is a complex and dynamic network system, and the perception of one’s body and external space involves the integration of different sensory information, which relies on the brain network composed of multiple brain regions and the synergy of multiple brain networks. Therefore, real-time evaluation of intra- and inter-brain network functional connectivity using resting-state fMRI (resting-state fMRI, rs-fMRI) has a certain value, and may better reflect the pathogenesis of VM. To exclude the influence of peripheral vestibular lesion as a confounding factor on brain functional changes, the present study included patients with VM without peripheral vestibular lesions from December 2018 to December 2020, constructed brain network connectivity model using independent component analysis, aimed to explore the altered sensory integration patterns in patients with VM in depth, thus providing a scientific theoretical basis for understanding whether VM is a no-structural central vestibular disease similar to persistent postural–perceptual dizziness, i.e., functional central vestibular disease with altered brain function.

## Methods

### Study subjects

A total of 17 patients with VM who received treatment in Aerospace Center Hospital, Peking University Aerospace School of Clinical Medicine between December 2018 and December 2020 were included. Clinical data of all patients were collected. Neurological and neuro-ophthalmological examinations, blood pressure, cardiac function, thyroid function tests, immune-related laboratory tests and lower limb electromyography were performed to exclude other medical diseases. Videonystagmography, caloric test, head impulse test, and vestibular-evoked myogenic potential stimulation were performed to exclude peripheral vestibular lesions. MRI was performed to exclude severe neurological diseases.VM was diagnosed based on Bárány Society's diagnostic criteria for VM (2012 version) [9].

17 age- and gender-matched healthy controls (HCs) were included, they had no history of headache or vertigo and had no severe medical diseases. 8 age- and gender-matched patients with migraine were also included in the study, patients with migraine were diagnosed according to the diagnostic criteria of ICHD-3rd edition (beta version) in 2013 [10], patients with migraine had no history of vertigo.

All subjects underwent peripheral vestibular function evaluation to exclude peripheral vestibular lesions. Patients with VM (during an inter-ictal phase), HCs, and patients with migraine (during an inter-ictal phase) were further scanned for fMRI. This study was approved by the Ethics Committee of Aerospace Center Hospital, Peking University Aerospace School of Clinical Medicine. All subjects volunteered to participate in this study and signed informed consent.

### Image acquisition

All subjects were scanned using a 3.0-Tesla MR (SIEMENS MAGNETOM Skyra syngo MR D13, Germany) with a 16-channel head and neck coil. During image acquisition, subjects' head was immobilized to avoid head movement. Subjects were asked to relax with their eyes closed, stay awake throughout the scanning. Images were obtained in sagittal plane using a 3D gradient-echo T1WI sequence with the following parameters: 192 sagittal slices; repetition time (TR) = 1900 ms; echo time (TE) = 2.43 ms; flip angle (FA) = 8°; field of view (FOV) = 256 mm × 256 mm × 256 mm; Voxel size = 1.0 mm × 1.0 mm × 1.0 mm. Resting-state fMRI images were obtained using echo-planar imaging (EPI) sequence sequence: TR = 2000 ms; TE = 30 ms; FA = 90°; FOV = 222 mm × 222 mm × 222 mm; Voxel size: 3.0 mm × 3.0 mm × 3.0 mm. After scanning, a total of 200 volumes were obtained, and the scan time was 6 min and 48 s. All subjects remained awake during scanning and did not experience significant discomfort during or after scanning.

### MRI image data processing

Image data were processed using DPARSFA software on the MATLAB2013 platform,

### Pre-processing of fMRI data

(1) data conversion: image data were converted from DICOM format to NIFTI format that can be processed by SPM12; (2) removal of the first 10 time points: due to the possible instability of the initial MRI signal caused by T1 relaxation and the need for participants to adapt to the scanning environment, the first 10 time points were deleted; (3) time correction: time correction is performed to ensure that all voxels within one volume had been acquired at the same time, the 35th slice was chosen as the reference slice; (4) head movement correction: slight head motion of the subject between the time points during the scan was corrected, to ensure the accuracy of the position information, the subjects who had more than 2.0 mm head translation in *x*-, *y*-, or *z* -direction and 1° head rotation were removed; (5) spatial normalization: to solve the problems related to difference in brain morphology among different subjects and the inconsistencies in spatial position during scanning, fMRI images were spatially normalized to a standard space using DARTEL and resampled at a resolution of 3 mm × 3 mm × 3 mm; (6) spatial smoothing: smoothing was conducted with a Gaussian kernel of 8 mm × 8 mm × 8 mm to reduce registration errors and increase the normality of the data.

### Independent component analysis

Independent component analysis was performed using GIFT software (Version 3.0a, http://mialab.mrn.org/software/gift/index.html). (1) Data reduction is conducted using principle component analysis; (2) the minimum description length (MDL) was used to estimate the number of independent components (ICs), the final estimated IC was 28; (3) the infomax algorithm was utilized for group independent component analysis. To ensure data repeatability, we performed the ICASSO for 100 times; (4) the IC time courses and spatial map of individual subjects were reverse reconstructed using spatial–temporal regression. After the reverse reconstruction, the time course of each participant's IC and space IC is acquired and the spatial map is converted into z-score.

### Intra-network functional connectivity

For each independent component of all subjects, one-sample *t* test was performed in patients with VM, HCs and patients with migraine, respectively, to create a sample-specific component map and a network mask. Multiple comparisons were corrected with false discovery rate (FDR) method (*P* < 0.05). Each mask of two groups was further combined into the total MASK of each component. A two-sample *t* test with regressing covariates, such as age and gender, was conducted to compare the differences in functional connectivity in each component within the corresponding network mask between patients with VM and HCs. The results of multiple comparisons were corrected by FWE (*P* < 0.05).

### Inter-network functional connectivity

For each subject, the time course of each network component is extracted to calculate the Pearson correlation coefficient (*r*) of each network component and other network time series, namely the functional network connectivity. The resulting r values were normalized into *Z* values by Fisher-*Z* transformation. Therefore, a 13 × 13 matrix was obtained. A two-sample *t* test was conducted to compare the differences in inter-network functional connectivity between patients with and HCs, as well as patients with migraine. The results of multiple comparisons were corrected by FDR (*P* < 0.05).

## Results

### Clinical data of patients included in the study

In the VM group, there are 7 males and 10 females, all were right-handed, and the average age was 39.47 ± 9.78 years. The median length of medical history was 5 years (range 1–30 years). As to the frequency of VM attacks, one patient reported one attack per week, 7 patients reported one attack per month, 9 patients reported once attack per year. In the HC group, there were 7 males and 10 females with an average age of 39.82 ± 13.01 years, all HCs were right-handed. In the migraine group, there were 3 males and 5 females, all were right-handed, the average age was 41.88 ± 11.03 years.

All 17 (100%) patients with VM had a history of migraine, including 12 (70.6%) patients of recurrent spontaneous vertigo, 2 (11.7%) patients of visually-induced vertigo, and 3 (17.6%) patients of head motion-induced vertigo. All 17 patients with VM reported worsening of dizziness during visual stimulation. The accompanying symptoms were photophobia or phonophobia (*n* = 15, 88.2%), migraine-like headache (*n* = 8, 47.1%), visual aura during VM attacks (*n* = 7, 41.2%).

During the caloric test, hyperactive response was found in 5 (29.4%) patients with VM, and caloric test intolerance was found in 12 (70.6%) patients. Among 17 patients with VM, 11 (64.7%) patients had a history of motion sickness (Table 1).

**Table 1:** Clinical characteristics of patients with vestibular migraine

Id/gender/age	Recurrent spontaneous vertigo	Accompanying migraine features			Caloric test		Motion sickness
		Photophobia /Phonophobia	Migraine-like headache	Visual aura	Hyperactive response	Caloric test intolerance	
1/F/52		√					
2/M/24	√	√	√	√		√	√
3/F/30	√	√					√
4/M/47	√	√				√	√
5/M/40	√	√				√	√
6/M/25		√	√		√	√	√
7/F/38	√	√	√	√		√	√
8/M/39		√			√	√	
9/F/35	√	√				√	
10/F/34		√	√	√	√	√	√
11/F/58	√		√				√
12/M/35	√	√				√	
13/F/47	√	√	√	√	√	√	√
14/F/54	√	√	√	√	√	√	√
15/F/44		√					
16/F/38	√	√		√			√
17/M/31	√		√	√		√	

^a^The criteria for hyperactive responses were as follows: total peak cool response (LC + RC) of > 99°/s, total peak warm response (LW + RW) of > 146°/s, total peak response (LC + RC + LW + RW) of > 221°/s. Caloric test intolerance refers to the main symptoms, including obvious nausea, vomiting, numbness in hands and feet, and body stiffness. Caloric test intolerance was considered severe if its duration was > 1 h

### Resting-state network components

The network components were identified in accordance with a previous study. Totally 13 independent components were obtained, including the salience network (SN); posterior default mode network (pDMN) that mainly includes posterior cingulate gyrus, precuneus and bilateral lateral parietal cortex; medial visual network (mVN), the primary visual cortex that contains occipital regions; lateral visual network (lVN), the higher-level visual cortex, that includes lateral part of the occipital lobe; occipital pole visual network (oVN); auditory network (AN) that is mainly composed of bilateral superior temporal gyrus, Heschl's gyrus and posterior insula; anterior default mode network (aDMN) that mainly contains medial prefrontal cortex and anterior cingulate cortex; sensorimotor network (SMN) that mainly involves includes sensorimotor cortex, supplementary motor areas (SMA), and secondary somatosensory cortex; sub-cortical network (SC); right frontoparietal network (RFPN) and left frontoparietal network (LFPN) that mainly include bilateral dorsolateral prefrontal cortex and posterior parietal cortex; executive control network (ECN) that primarily includes bilateral dorsolateral prefrontal cortex, intraparietal sulcus, inferior parietal lobule, precuneus, dorsal frontal lobe and middle cingulate cortex; cerebellar network (CN).

### Altered intra-network functional connectivity in patients with VM

**Fig. 1:**
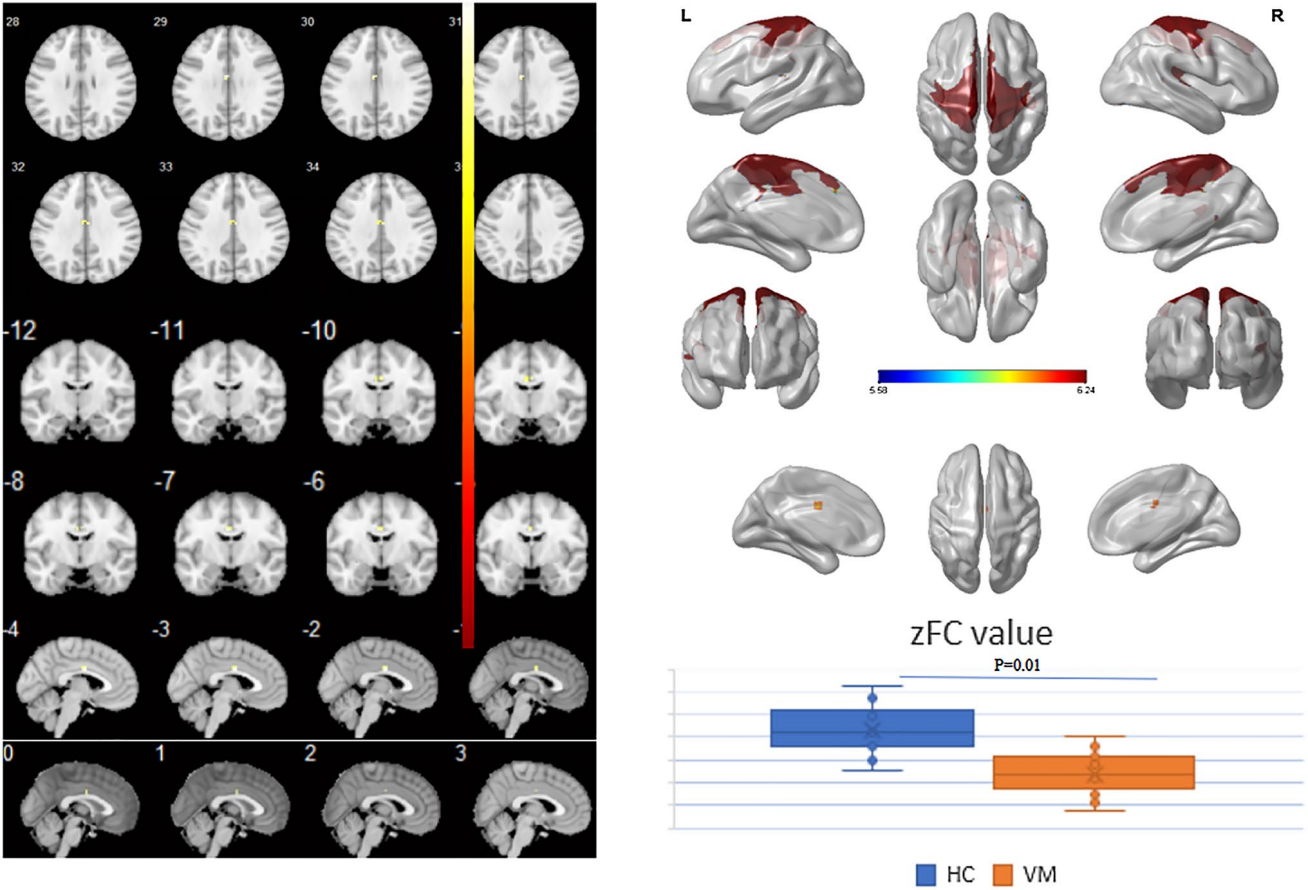
Comparison of differences between intra-network functional connectivity between patients with vestibular migraine and healthy controls. Compared with healthy controls, the functional connectivity in the bilateral medial cingulate gyrus and paracingulate gyrus within the sensorimotor network (SMN) was decreased (*X* = − 3, *Y* = − 6, *Z* = 30, *K* = 7, *P* < 0.05, FWE corrected)

The 13 network components were analyzed by analysis of variance (ANOVA) to compare the difference in intra-network functional connectivity between patients with VM and HCs. Compared with HCs, patients with VM showed decreased functional connectivity in the bilateral medial cingulate gyrus and paracingulate gyrus within SMN (*X* = − 3, *Y* = − 6, *Z* = 30, *K* = 7, *P* < 0.05, FWE corrected, Fig. 1).

### Altered inter-network functional connectivity in patients with VM

**Fig. 2:**
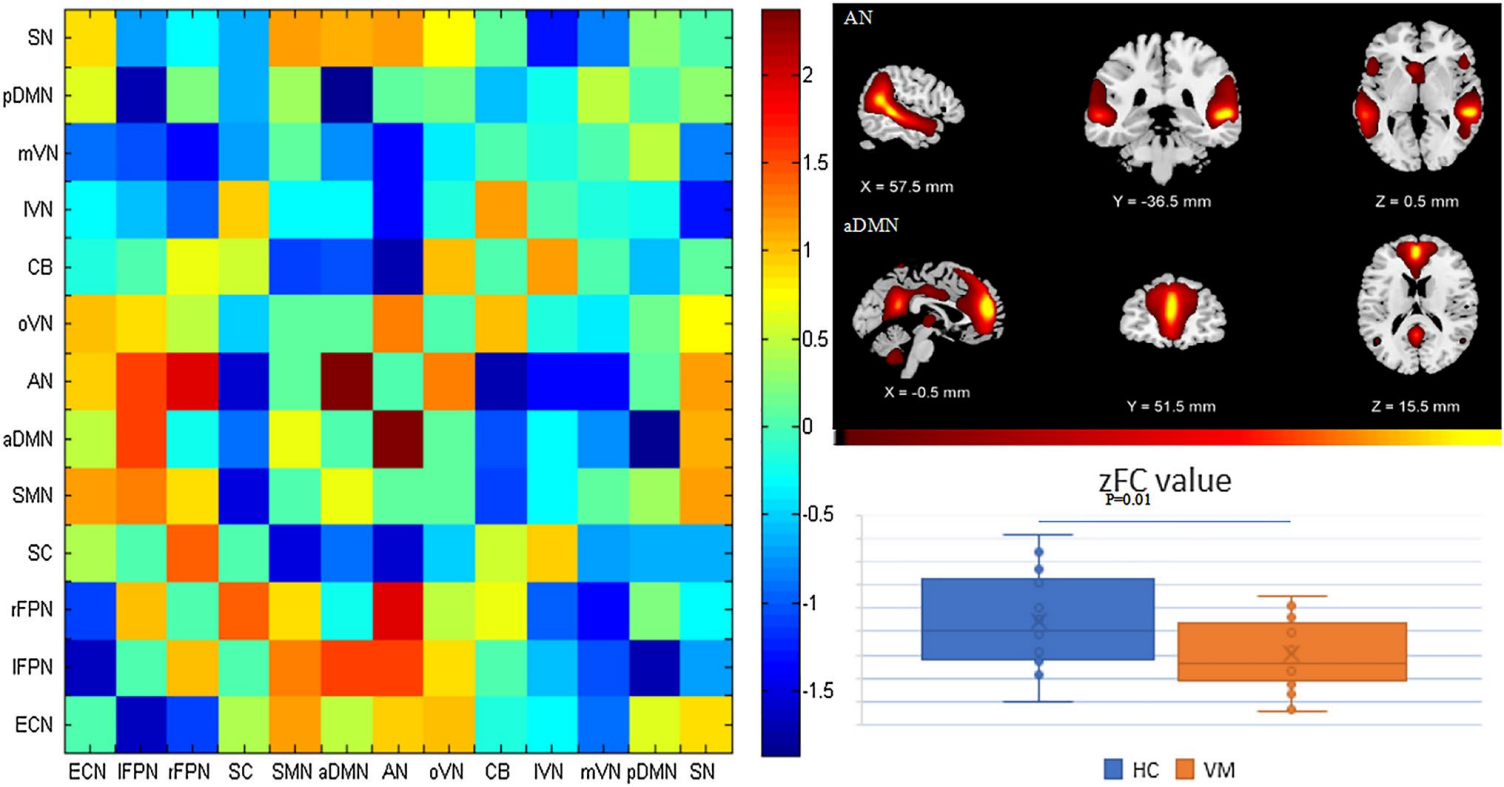
Comparison of differences between inter-network functional connectivity between patients with vestibular migraine and healthy controls. Compared with healthy controls, the functional connectivity between auditory network (AN) and anterior default mode network (aDMN) was weakened (*P* < 0.05, FWE corrected)

**Fig. 3:**
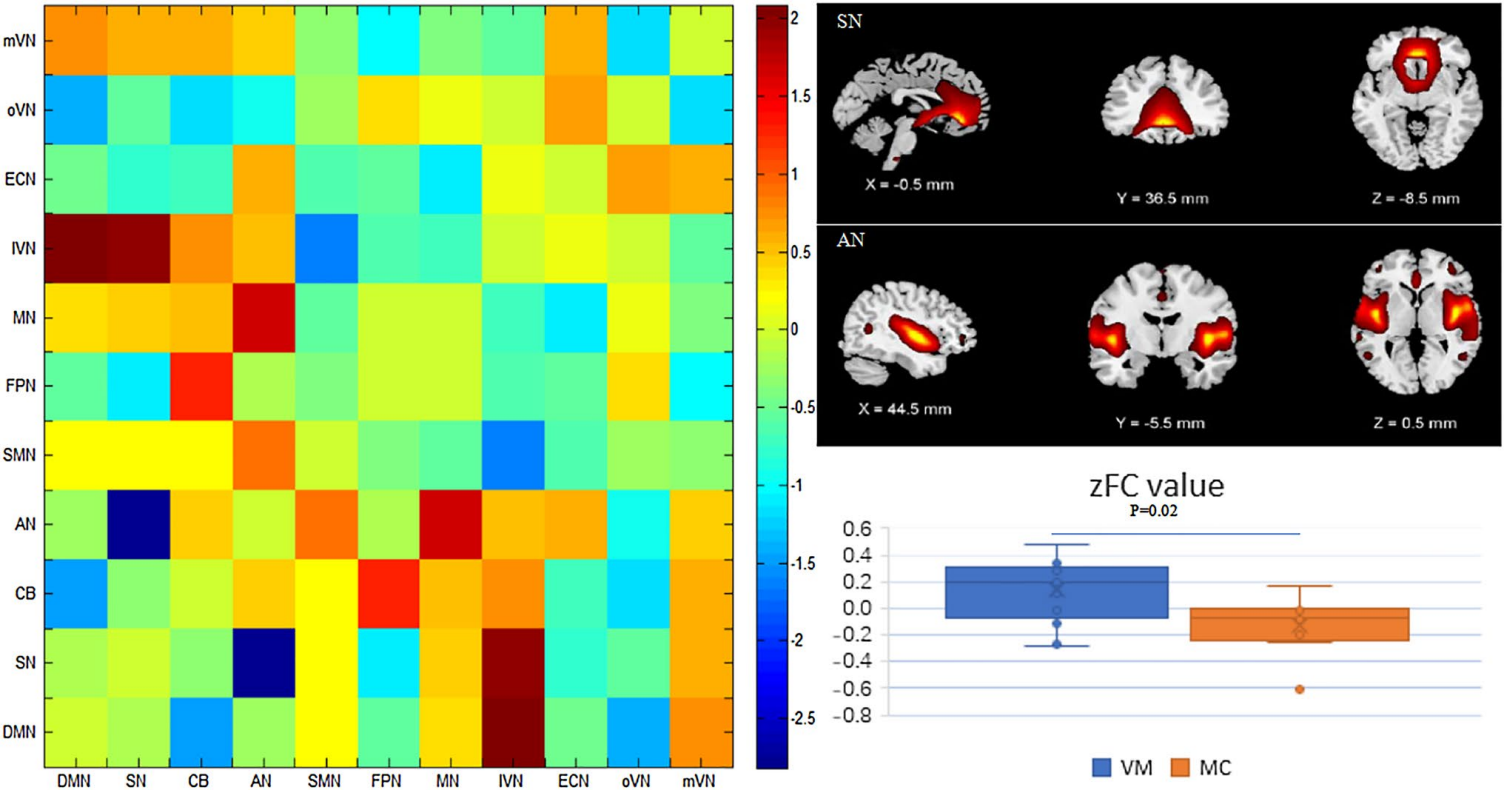
Comparison of differences between inter-network functional connectivity between patients with vestibular migraine and those with migraine. Compared with patients with migraine, the functional connectivity between auditory network (AN) and salience network (SN) was enhanced (*P* < 0.05, FWE corrected)

The difference in inter-network functional connectivity was compared between patients with VM and HCs, as well as patients with migraine. Patients with VM showed weakened functional connectivity between AN and aDMN compared with HCs (*P* < 0.05, FWE corrected), and enhanced functional connectivity between AN and SN compared with patients with migraine (*P* < 0.05, FWE corrected, Figs. 2, 3).

## Discussion

### Decreased functional connectivity in the median cingulate and paracingulate gyri (DCG) within SMN in patients with VM

Previous studies [4, 5] have demonstrated the involvement of vestibular–thalamic–cortical pathway (such as bilateral cerebellum, frontal and temporal cortices, posterior insula, and thalamus) in patients with VM during an ictal period; however, there is a lack of studies that investigated the independent components of resting-state brain network in VM patients during an inter-ictal period. In the present study, 13 independent components were identified. By comparing the difference in intra-network functional connectivity between patients with VM, HCs and patients with migraine, the results showed that compared with HCs, patients with VM showed decreased functional connectivity in the bilateral medial cingulate gyrus and paracingulate gyrus within SMN. As we all know, multi-sensory integration and sensorimotor integration are important aspects of regulating higher vestibular cognitive function at the level of the cerebral cortex. SMN includes SMA, sensorimotor cortex, and secondary somatosensory cortex. The SMN components are closely related to brain activation observed during bimanual movements, and it is also the first resting-state network identified using fMRI [11], which can form action–execution and perception–proprioception network patterns. Zhang et al. [12] found that during tasks requiring motor response inhibition, SMN extended to DCG. The cingulate gyrus is an important region for motor and vestibular processing [13]. Aron et al. [14] showed that DCG is a key region for proactive motor control, but not reactive motor control, which can be activated by increased endogenous neural activity evoked by stimulation. It is worth noting that Hoffstaedter et al. [15, 16] found that higher-order DCG activity was observed when subjects were asked to perform dual tasks, e.g., deciding which hand will perform the movement and the timing of movement. In addition, DCG also re-allocates attentional resources based on task-related information by monitoring conflicts in information processing [17]. According the above-mentioned findings, the DCG in healthy individuals is involved in conflict monitoring to facilitate task-related actions, and this neural network is non-dominant, but is necessary to achieve normal movement and spatial decision-making [18, 19]. In this study, patients with VM showed decreased functional connectivity in the DCG within SMN. We speculated that patients with VM have reduced function of monitoring motor network and spatial task-related actions, and their sensory–motor and vestibular cortex areas may be in a sensitive state.

### Altered functional connectivity between AN and aDMN as well as SN in patients with VM

We further compared the differences in inter-network functional connectivity between patients with VM, HCs and patients with migraine, and found that compared with HCs, patients with VM showed weakened functional connectivity between AN and aDMN. And compared with patients with migraine, patients with VM showed enhanced functional connectivity between AN and SN. No matter which group being compared with (HCs vs VM, or migraine vs VM), the AN was both a region of interest. AN primarily participates in the processing of auditory information and is mainly distributed in the cortex around the Sylvian fissure. A recent study conducted by Lopez et al. [20] showed that temporal and parietal lobes around the Sylvian fissure are the regions for auditory–vestibular integration, and are the core parts of the multisensory vestibular cortex. Studies [21–23] have found that superior temporal sulcus, temporo-parietal junction and the area around the intraparietal complex receive multi-sensory afferents. Auditory and vestibular information converge in the overlapping areas of the caudal part of the superior temporal sulcus and posterior insula, the caudal part of the superior temporal sulcus and posterior insula contain areas of vestibular contribution to auditory processing, i.e., higher vestibular cortices provide multisensory integration, this is important for tasks such as spatial localization of sound [24].

The DMN was discovered by Raichle et al. [25], which is a network of brain regions that are active when the brain is in the resting state and inactivated in the task state, i.e., endogenous and spontaneous functional brain activities are present when there is no task and the brain is at quiet and wakeful rest state, and these spontaneous brain activities may be closely associated with human emotions, thoughts and self-cognitive process [25, 26]. DMN is the one of the most prominent networks to be discussed in resting-state fMRI studies, and the independent components of DMN are the most widely studied There are many speculations about the basic functions of DMN. DMN mainly contains medial prefrontal cortex, posterior cingulate cortex, precuneus, anterior cingulate cortex, bilateral inferior parietal lobule and medial temporal lobe [24]. Among these brain regions, the medial prefrontal cortex and anterior cingulate cortex belong to aDMN, and the remaining regions, such as posterior anterior cingulate cortex, precuneus, and bilateral angular gyrus belong to pDMN.

**Fig. 4:**
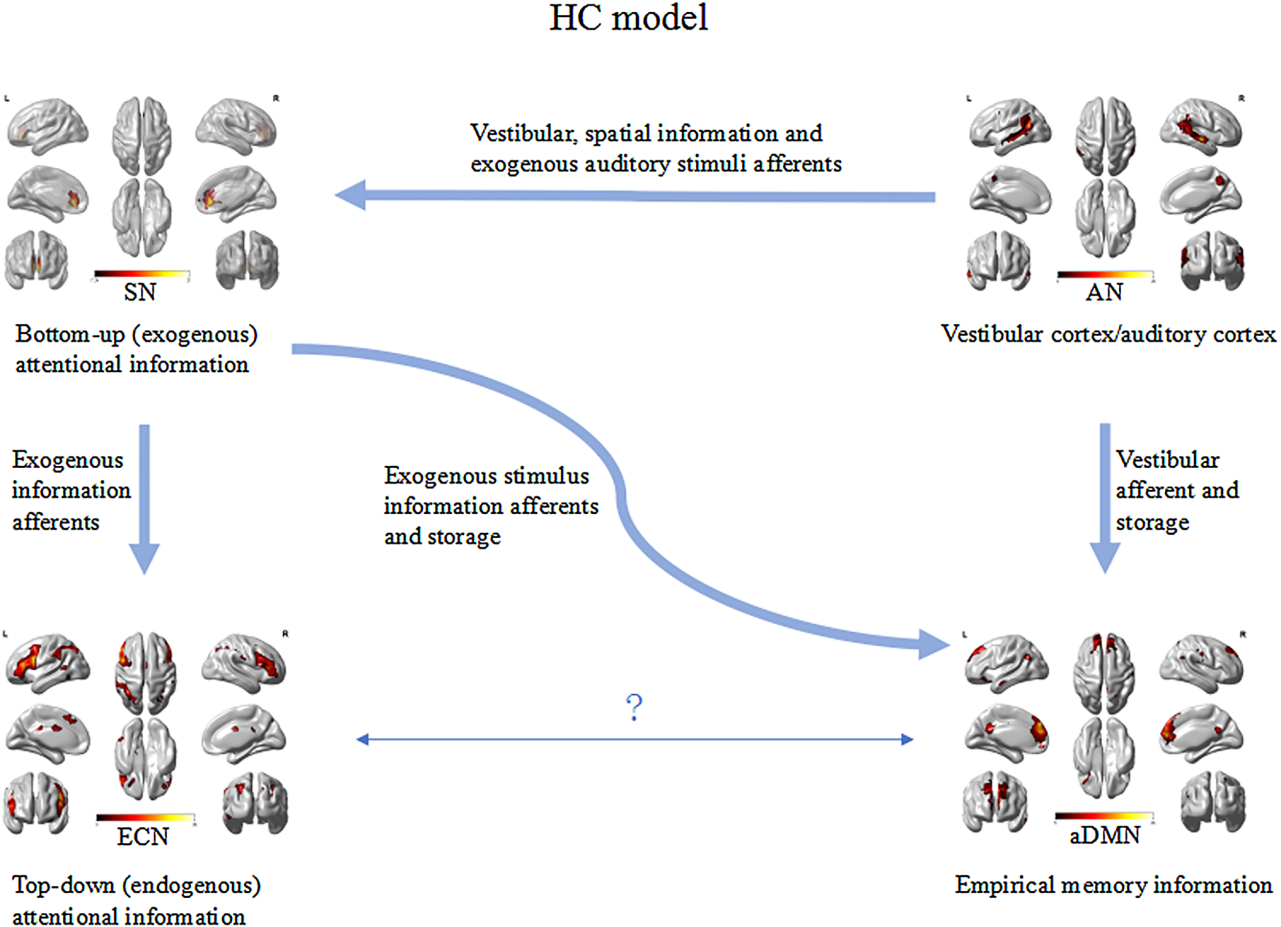
Functional connectivity between visual network (VN) and salience network (SN)-executive control network (ECN)-default mode network (DMN) in healthy controls

**Fig. 5:**
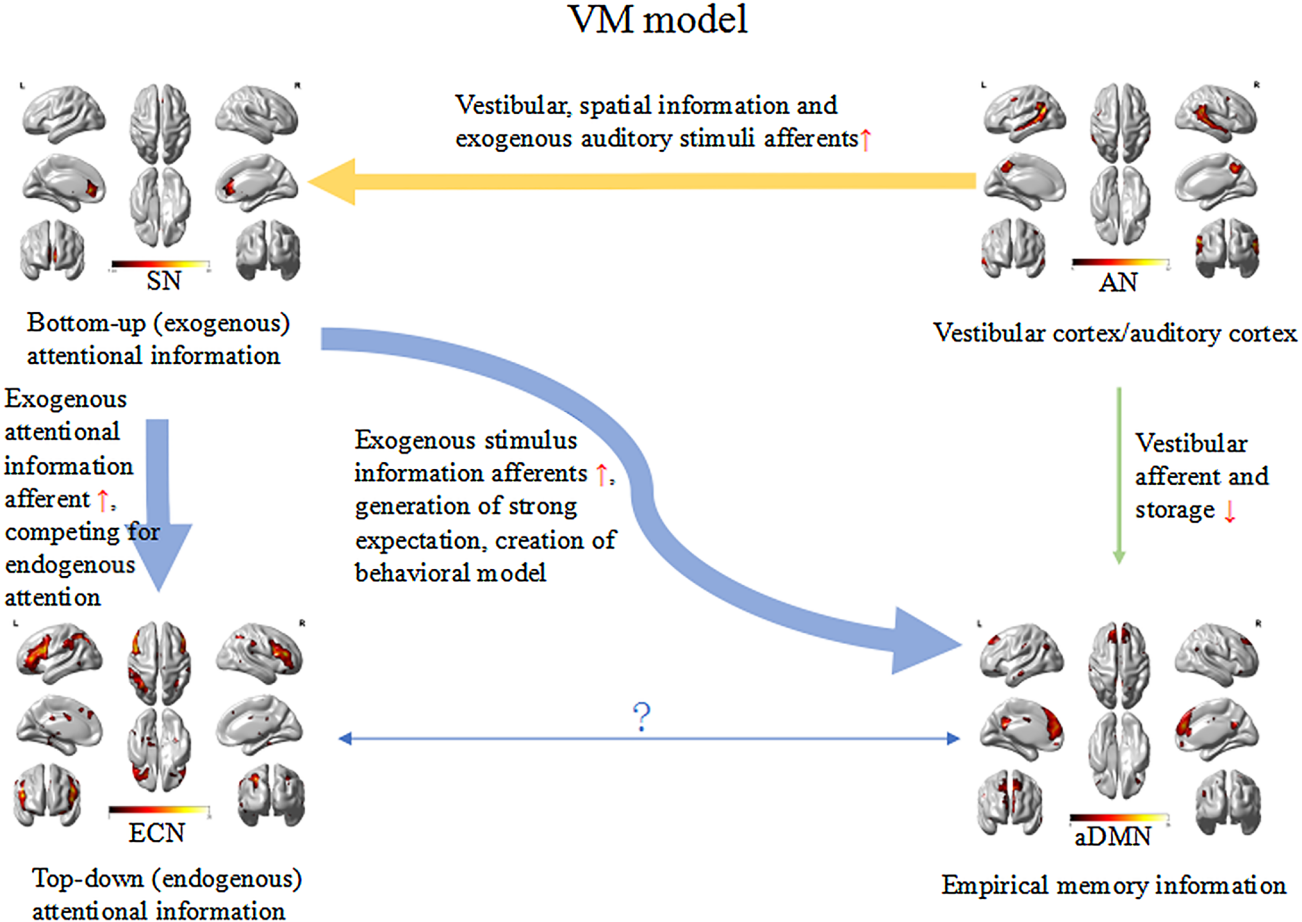
Functional connectivity between visual network (VN) and salience network (SN)-executive control network (ECN)-default mode network (DMN) in patients with VM. The yellow arrow indicates that functional connectivity between AN and aDMN was weakened in patients with VM compared with healthy controls. Green arrow indicates that functional connectivity between AN and SN was enhanced in patients with VM compared with patients with migraine

The SN is mainly composed of the anterior insula and the dorsal anterior cingulate gyrus, and has strong connections to the amygdala, striatum, and substantia nigra/ventral tegmental area [27]. The SN can quickly monitor the stimuli that are most relevant to one's own homeostasis and transfer them to the area responsible for target-related information processing, thereby modulating the bottom–up (exogenous) attentional information afferents [28, 29]. The areas that receive relevant stimuli from the SN are mostly located in the parieto-insular vestibular cortex area, including the precuneus, parietal lobe, temporal area, temporo-parietal junction, and ventromedial prefrontal cortex [29]. Sensory processing sensitivity may depend on the activity and interaction of three brain networks, SN, DMN, and ECN [32] (Fig. 4). The ECN primarily includes dorsolateral prefrontal cortex and posterior parietal cortex, and is responsible for allocating top–down (endogenous) attentional resources to stimuli that are consciously determined by individuals in the context of goal-directed behavior [30]. ECN is in a competitive relationship with SN that allocates exogenous attention [31]. In fact, the brain is not in a dormant state waiting for stimulus, but constantly generating expectation of the environments [32]. Expectation creates a behavioral model that allows the brain to devote relatively few resources to action [32], but devote additional cognitive and functional resources to unexpected environments (such as new vestibular stimuli) [33]. Assuming that the increased reactivity of the SN of sensitive individuals affects the previous vestibular information afferents, strong expectations are initially established, which are stored in the DMN [34–36]. The brain would expect that the new vestibular stimuli will be similar to those already stored (both in degree and type). However, if the stimuli do not match or exceed expectations, the spontaneous activity within the SN and the flow of information from the SN to the DMN will increase to gather more information to drive goal-directed behavior. If necessary, the SN will increase the connection with the ECN to allow it to redirect its attention to the external stimulus. During this process, the information flow in sensitive individuals may increase, because enhanced connection between SN and ECN allows individuals to redirect their attention when exposed to novel information (Fig. 5).

We speculated that patients’ experience of repeated dizziness during the ictal period of VM may be stored in the DMN [34–36], and during inter-ictal phase, vestibular stimuli experienced by patients with VM may be lower than the expectation stored in the DMN. Therefore, our findings showed that functional connectivity between AN and aDMN was decreased in patients with VM compared with HCs. A study showed that in comparison with patients with migraine, the vestibular pathway may be in a sensitive state in patients with VM during the inter-ictal phase. Our study documented enhanced functional connectivity between AN and SN in patients with VM compared with patients with migraine, this may presumably lead to abnormal integration of auditory and vestibular afferents in the SN-ECN-DMN network, with reduced stimulus threshold for the perception of vestibular information (Fig. 5).

In comparison with HCs, patients with VM showed decreased functional connectivity in the DCG, this may cause the SMN to be in a sensitive state. And since vestibular information afferent to the DMN was reduced during the inter-ictal period of VM, it is speculated that the DMN may store prior information about repeated dizziness during the ictal period of VM. In comparison with patients with migraine, patients with VM during the inter-ictal period showed enhanced functional connectivity between AN and SN, this may lead to reduced threshold for the perception of vestibular information.

## Limitations

The limitation of the study is the small sample size; further study with larger sample size is needed to further conform our results. Our findings provide evidence for functional brain network properties in patients with VM and for further functional connectivity studies based on ROI of multilevel vestibular pathways, as well as brain network connectome studies based on small-world brain graph theory.

## Conclusion

Patients with VM showed obvious altered functional connectivity in the bilateral medial cingulate gyrus and paracingulate gyrus within the SMN. The DCG may be impaired in these patients, the disinhibition in the sensorimotor network and vestibular cortical network may result in hypersensitivity phenomena, such as photophobia, phonophobia. Altered functional connectivity between AN and DMN, SN in patients with VM may lead to increased sensitivity to vestibular sensory processing.
